# Smoking, Blood Pressure, and Cardiovascular Disease Mortality in a Large Cohort of Chinese Men with 15 Years Follow-up

**DOI:** 10.3390/ijerph15051026

**Published:** 2018-05-18

**Authors:** Jibin Tan, Xiumin Zhang, Weihua Wang, Peng Yin, Xiaomin Guo, Maigeng Zhou

**Affiliations:** 1 School of Public Health, Jilin University, Changchun 130021, China; tanjb@chinacdc.cn; 2 Chinese Center for Diseases Control and Prevention, Beijing 102206, China; guoxm@chinacdc.cn; 3 Shaanxi Provincial Center for Disease Control and Prevention, Xi’an 710054, China; wwh1220066@126.com; 4 National Center for Chronic and Noncommunicable Disease Control and Prevention, Beijing 100050, China; yinpeng@ncncd.chinacdc.cn (P.Y.); zhoumg@chinacdc.cn (M.Z.)

**Keywords:** blood pressure, smoking, mortality, cohort

## Abstract

Background: To examine the joint effects of smoking and blood pressure on the risk of mortality from cardiovascular disease (CVD) in a cohort of Chinese men. Methods: This study followed a cohort of 213,221 men over 40 years of age who were recruited from 45 district/counties across China between 1990–1991, and whose cause-specific mortality was examined for 15 years, up to 31 December 2005. We calculated hazard ratios for all-cause mortality and CVD, ischemic heart disease (IHD), and stroke mortality for the combined sets of smoking status and blood pressure levels using the Cox proportional hazard model, adjusting for potential individual-level and contextual-level risk factors. Results: During the 15 years of follow-up, 52,795 deaths occurred, including 18,833 deaths from CVD, 3744 deaths from IHD, and 11,288 deaths from stroke. The risk of mortality from CVD, IHD, and stroke increased significantly, with increased systolic blood pressure (SBP), diastolic blood pressure (DBP), and with more pack years of smoking. Compared with never-smokers with normal blood pressure, the hazard ratios and 95% CI of CVD, IHD, and stroke mortality for those who smoked over 20 pack years with hypertension were remarkably increased to 2.30 (95% CI: 2.12–2.50), 1.78 (95% CI: 1.48–2.14), and 2.74 (95% CI: 2.45–3.07), respectively. Conclusion: There was a combined effect on the risk of CVD, IHD, and stroke mortality between smoking and hypertension. The joint efforts on smoking cessation and lowered blood pressure should be made to prevent cardiovascular disease mortality in Chinese men.

## 1. Introduction

Cardiovascular disease (CVD) is a leading cause of mortality and morbidity [1]. The most recent Global Burden of Disease (GBD) Study estimated that in 2016, 17.6 million deaths were caused by CVD worldwide, which increased by 14.5% between 2006–2016 [1]. Globally, smoking and high systolic blood pressure are the two leading risk factors in terms of attributable disability-adjusted life years (DALYs) for men, causing 124.1 million DALYs and 122.2 million DALYs respectively in 2016, based on the GBD 2016 estimates [2]. In China, stroke and ischemic heart disease (IHD) were ranked as the first and second leading causes of total years of life lost (YLL) in 2016 [2]. The prevalence of smoking among adult Chinese men remained at high levels, Chinese men aged over 18 years comprising 54.0% of the current smokers in 2010 [3]. The prevalence of hypertension has been also increasing rapidly in the urban and rural areas in China in recent years [4,5].

The positive association between blood pressure and CVD has been well documented in many prospective cohort studies in both developed and developing countries [6,7,8,9]. Smoking is also associated with blood pressure level and CVD mortality [10,11]. Although strong evidence supports that smoking and hypertension act on CVD mortality risk, the combined effects of these factors have been rarely studied, especially in China, where the prevalence of smoking and hypertension are both high. 

We report data from a prospective cohort study including more than 220,000 Chinese men aged 40 years and above who were recruited from 49 urban and rural areas in China between 1990–1991, and followed up for mortality up until 2006. This paper aims to examine the joint effects of smoking and hypertension, both overall and within different population subgroups, on the risk of subsequent CVD mortality.

## 2. Materials and Methods

### 2.1. Study Population

The design of the cohort has been described elsewhere [12]. Briefly, 228,459 men over 40 years old were recruited from 49 district/counties across China in 1990 and 1991, which were randomly selected from China’s 145 disease surveillance points (DSPs). DSPs are nationally representative and cover about 1% of China’s total population. A DSP site was either a district in an urban area or a county in the rural area. In each district/county, two or three residential units were randomly selected, and all of the resident men older than 40 years were invited to participate in the study, of whom about 80% accepted. The baseline survey included a standardized questionnaire and physical measurements that were administered by trained health workers to obtain information on demographics, lifestyle factors (smoking, alcohol drinking, and household solid fuel use), personal medical history, height, and weight. Vital status was monitored by local DSP staff through death registries, which took place alongside regular crosschecks from local residential records kept at the Public Security Department and the Social Welfare Department; these were supplemented by annual active confirmation by local residential committees. The underlying cause of death was ascertained from official death certificates, which were supplemented with information from medical records and coded according to International Classification of Diseases version 9 (ICD-9). Cohort follow-up was from 1990/1991 to 2006. CVD-related death (ICD-9 390–414 and 420–459, including IHD and stroke, as well as chronic rheumatic heart diseases, hypertensive heart disease, hemorrhagic stroke, ischemic stroke, subarachnoid hemorrhage, and pulmonary heart disease), which was defined as death from ischemic heart disease (IHD) (ICD-9 410–414) and stroke (ICD-9 431–439) was specifically examined in the present study. This study received approval from the ethics committee from Chinese Center for Disease Control and Prevention.

### 2.2. Definition of Hypertension

Hypertension was defined if the participant was diagnosed by a health professional or took any antihypertensive drugs in the last two days before the interview, or if their measured systolic blood pressure (SBP) ≥140 mmHg or diastolic blood pressure (DBP) ≥90 mmHg for those who did not take any antihypertensive drugs or was not diagnosed by a health professional. To examine the sensitivity of blood pressure, we further categorized blood pressure into three clinical groups according to 2010 Chinese guidelines for the management of hypertension [13]. Clinical groups were categorized as normal, SBP < 120 mmHg and DBP < 80 mmHg; prehypertension, 120 mmHg ≤ SBP ≤ 139 mmHg or/and 80 mmHg ≤ DBP ≤ 89 mmHg; and hypertension, SBP ≥ 140 mmHg or/and DBP ≥ 90 mmHg.

### 2.3. Definition of Variables

Covariates in the study were defined as marital status (married, not married), educational level (≤6 years or >6 years of education), smoking status (never, pack years of cigarettes smoked (one pack contains 20 cigarettes)), alcohol drinking (non-regular drinkers, 0–17 drinks (1 drink equals 14 g of pure alcohol)/week, 18–35 drinks/week, >35 drinks/week), body mass index (BMI) (kg/m^2^), consumption of fresh fruit and vegetables, indoor air pollution defined as household solid fuel use for heating or cooking (no, yes), urbanity (urban/rural), region (north, south). Urban was a district in a city, and rural was a county in the baseline recruitment according to criteria by China National Statistics Bureau.

### 2.4. Statistical Analysis

Continuous variables are shown as the mean (standard deviation), and categorical variables are shown as numbers and percentages. To examine the combined effects of smoking and blood pressure, participants were categorized into six different sets according to smoking status (never, 0–19 pack years smoked, and ≥20 pack years smoked) and blood pressure levels (normal, prehypertension, and hypertension). We used Cox proportional hazard models for survival, with the time scale setting as duration from year of recruitment to the year of death, or censored at the year as outcome, and SBP or DBP at baseline as the exposure factors for the main analysis.

To evaluate the effect of different confounding factors, we used three models to assess the association between blood pressure and mortality from all-cause, CVD, ischemic heart disease (IHD), and stroke. Variables and covariates for inclusion in the models were based on several previous studies, as well as the authors’ interest [14,15]. In model one, we only adjusted ages at entry. In model one, we further added individual-level risk factors such as marital status, educational level, smoke status, pack years of cigarettes smoked, alcohol drinking, units of alcohol per week, BMI (kg/m^2^), consumption of fresh fruit and vegetables, and indoor air pollution. In model three, we further included urbanity (urban versus rural) and region (south versus north) as contextual variables, to adjust for broad-scale spatial variation in mortality not accounted for by the risk factors that were included in the survival model. For each model, the mortality hazard ratio (HR) and 95% confidence intervals (95% CI) associated with a 10-mmHg increase in mean SDP or DBP, as well as categories of blood pressure (normal, prehypertension, hypertension), were estimated. The HR and 95% CI were also calculated for the risks of all-cause, CVD, IHD, and stroke mortality according to different smoking status, smoking packs per day, pack years of cigarettes smoked, and years of smoking. Joint effects of blood pressure and smoking on disease outcomes were plotted using never-smokers with normal blood pressure as the reference group. Analyses were performed using SAS version 9.4 and R version 3.0.1.

## 3. Results

### 3.1. General Information of the Participants

**Figure 1:**
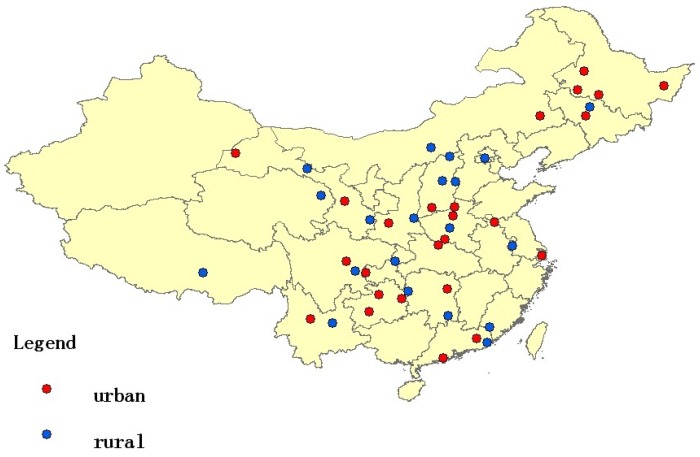
Geographic locations of the 45 cohort sites.

The geographic locations of the 45 cohort sites are shown in Figure 1. Descriptive analysis of the participants at baseline is presented in Table 1. Of the 213,221 participants, 58,583 (27.5) were never-smokers, and 102,747 (48.2) smoked more than 20 pack years of cigarettes. The mean age at baseline was 54.7 years, and the mean BMI was 21.8 kg/m^2^. The majority (71.1%) of the participants lived in rural areas, and 45.9% were in the north. Most (66.4%) of the participants had an educational level of less than six years. Participants who smoked more than 20 pack years tended to be heavy drinkers who were less educated, lived in rural areas, were more exposed to indoor air pollution, and had less consumption of fruit and vegetables.

### 3.2. Association between Blood Pressure and Outcomes

In total, 52,795 deaths occurred during the 15 years of follow-up. These included 18,833 deaths from CVD, 3744 deaths from IHD, and 11,288 deaths from stroke. As shown in Table 2, a 10-mmHg increase in SBP or DBP based on average blood pressure during 1990–1991 was associated with increased risk of mortality from all causes, CVD, IHD, and stroke in the model adjusted for age or individual-level covariates. In the fully adjusted model that include both individual and area-level covariates, we observed that a 10-mmHg increase in SBP was associated with a higher mortality risk for all causes (heart rate (HR) 1.07, 95% CI 1.06, 1.07), CVD (HR 1.16, 95% CI 1.15, 1.17), IHD (HR 1.08, 95% CI 1.06, 1.10), and stroke (HR 1.20, 95% CI: 1.19, 1.21). The pattern on DBP was similar, but the effect size was consistently higher than that of SBP. When blood pressure was grouped as normal, prehypertension, and hypertension, the effect size of hypertension was higher than prehypertension in each model, and regarding outcomes, hypertension was associated with the highest mortality risk for stroke.

### 3.3. Association between Smoking Status and Outcomes

As shown in Table 3, the *p*-values for trend were all <0.0001. Compared with never-smokers, the hazard ratios of pack years were significantly higher for all-cause (HR 1.21, 95% CI 1.18–1.24), CVD (HR 1.14, 95% CI 1.10–1.19), IHD (HR1.22, 95% CI 1.13–1.33), and stroke (HR 1.14, 95% CI 1.08–1.19) mortality. Similar results were observed in associations between smoking packs per day, smoking status, and years of smoking.

### 3.4. Association between the Joint Effects and Outcomes

**Figure 2:**
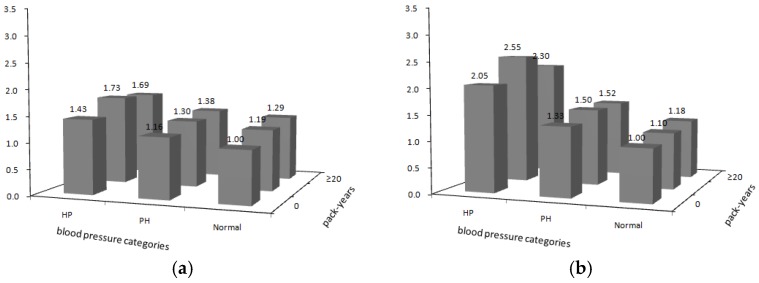

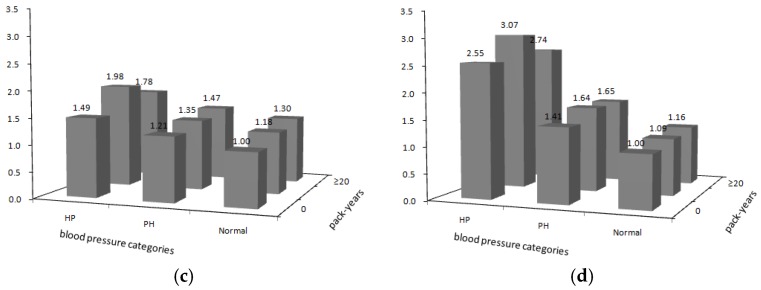
Joint effects of smoking and blood pressure level on the risk of deaths ((**a**) all-cause; (**b**) CVD; (**c**) IHD; (**d**) Stroke). Hazard ratios against blood pressure levels (normal = normal blood pressure, PH = prehypertension, HP = hypertension) and pack years of smoking (0, 0.1–19, ≥20) adjusted for age, educational level, marital status, indoor air pollution, consumption of fruit and vegetables, alcohol drinking, urbanicity, and region).

The four graphs in Figure 2 present the joint effects of smoking and blood pressure on the risk of mortality from the aforementioned four causes of death. There were significant increasing mortality risks with increasing blood pressure levels for all four causes among never-smokers, smokers who smoked 0–19 pack years, and smokers who smoked more than 20 pack years. For participants with normal blood pressure and prehypertension, the risks increased significantly with more pack years smoked compared with never-smokers. For hypertensive participants, although smokers were at a higher mortality risk compared with never-smokers, the hazard ratios for smokers with 0–19 pack years were slightly higher than smokers with more than 20 pack years, and this pattern was observed for all four disease outcomes. Compared with never-smokers with normal blood pressure, the hazard ratios and 95% CI of CVD, IHD, and stroke mortality for those who smoked over 20 pack years with hypertension were remarkably increased to 2.30 (95% CI: 2.12–2.50), 1.78 (95% CI: 1.48–2.14), and 2.74 (95% CI: 2.45–3.07), respectively. Tables S1–S4 present the HRs and 95% CI for the joint effects of smoking and blood pressure on the risk of all cause, CVD, IHD and stroke mortality respectively. To show the effects of all potential confounding factors, we present the detailed model results including HRs and 95% CI for all variables in the adjusted model. Please see Appendencies in supplementary materials. 

## 4. Discussion

Our study indicates that smoking and blood pressure are important risk factors for CVD and all-cause mortality. A significant association between smoking exposure and blood pressure and the risk of all-cause and CVD mortality was observed, after adjustment for potential risk factors. The combination of pack years smoked and high blood pressure category was associated with much higher risk of all-cause and CVD mortality. A particularly high effect size was observed for stroke mortality.

The joint effect of smoking and blood pressure on CVD mortality have been investigated in previous studies. A large cohort found that there existed a combined interaction on an additive scale between current smoking and high SBP (140 mmHg) for the risk of IHD in the female adult Chinese population [14]. The significant relationship between blood pressure and CVD mortality, and the joint effects of smoking and blood pressure observed in our study were in agreement with previous studies [15,16]. The effect size was also similar compared with previous studies on the Chinese population, but was lower than studies on Western populations, which could be due to different smoking exposure doses or the profiles of lipid levels [17,18,19]. We observed a consistently higher effect size for stroke than IHD, suggesting the importance of smoking cessation in actions reducing the burden of stroke, which is the leading cause of death according the most recent GBD estimates [1].

A pooled analysis from three large-scale cohort studies in Japan suggested that smoking cessation led to a risk reduction in mortality from cardiovascular disease, regardless of the duration of smoking or the number of cigarettes smoked per day [10]. Evidence from another Chinese cohort not only confirmed the significant reduction of IHD and stroke mortality associated with smoking cessation, but also indicated that the benefits of smoking cessation were underestimated in previous studies that did not use repeated measures [20]. It is noteworthy that we observed a slightly higher hazard ratio for smokers with 0–19 pack years than smokers who had smoked over 20 pack years in hypertensive participants. This was in accordance with another Chinese cohort study that reported a higher risk of CVD mortality in the 0.1–9 pack-years group than those smoked 10–19 and ≥20 pack years [15]. This may indicate that the additive effects of smoking may attenuate when hypertension has been developed. Further in-depth research is needed in order to address this non-linear association between smoking amount and CVD mortality risks among hypertensive patients. 

Smoking as the most important risk factor for many chronic diseases, including CVD, has been well documented [2,3]. The prevalence of smoking remained at high levels since the 1990s in China, with the smoking rate as high as 53% for adult Chinese men. Smoking caused about 20% of all adult male deaths in China during the 2010s, and it is estimated that the annual number of deaths in China that are caused by tobacco will rise from about one million in 2010 to two million in 2030 and three million in 2050, unless there is widespread cessation [21]. Our findings lend support to more comprehensive and stringent smoking cessation programs targeting Chinese men. 

Our study has many strengths. We used a prospective cohort study design with stringent quality control procedures. The sample size was large, with approximately 220,000 men included with a very high follow-up rate. The follow-up was 15 years, allowing sufficient endpoint events and the outcome measurements are accurate based on the well-developed DSP system. The standardized comprehensive questionnaires at baseline allowed us to adjust for most of the confounding factors such as educational level, BMI, fruit and vegetable consumption, alcohol drinking, and indoor air pollution from solid fuel combustion. Due to the large sample size, we were also able to adjust for some contextual factors, such as urbanicity and geographic region. 

Our study also has some limitations. Firstly, we only included men in our cohort and could not examine the joint effect of smoking and blood pressure in women. However, the prevalence of smoking among Chinese women is less than 3%, and has remained at very low levels over the last two decades. The smoking-caused burden of disease is most likely to be significant in men, as illustrated in the present study. Secondly, we were not able to adjust for some important risk factors such as physical activity, serum lipid, and glucose levels due to the data availability. We therefore cannot exclude the possibility that these factors may modify the effect of smoking and BP on CVD mortality. Thirdly, we only had information on smoking status and blood pressure at baseline, without further data on how smoking status changed during follow-up, which prevented us from performing any reliable follow-up analyses on the relationship between smoking status and BP.

In conclusion, we found a combined effect of smoking and blood pressure levels on the risk for CVD, IHD, and stroke mortality in Chinese men. While many programs have been carried out to reduce smoking prevalence and lower blood pressure, joint sustainable efforts on smoking cessation and hypertension management should be made to prevent cardiovascular disease mortality in China. 

## Figures and Tables

**Table 1:** Baseline characteristics of study population in the cohort according to pack years smoked.

Characteristics		Pack Years Smoked		
	*n* (%)	0	0.1–19	20
*n* (%)	213,221	58,583 (27.5)	51,891 (24.3)	102,747 (48.2)
Age at enrollment (years), mean ± SD	54.7 ± 10.6	55.4 ± 11.1	51.3 ± 9.7	56.1 ± 10.4
Marital status				
No	18,895 (8.9)	6286 (10.7)	3518 (6.8)	9091 (8.9)
Yes	194,326 (91.1)	52,297 (89.3)	48,373 (93.2)	93,656 (91.1)
Educational level				
<6 years	141,610 (66.4)	36,263 (61.9)	31,662 (61.0)	73,685 (71.7)
≥6 years	71,611 (33.6)	22,320 (38.1)	20,229 (39.0)	29,062 (28.3)
BMI (kg/m^2^), mean ± SD	21.8 ± 2.7	22.1 ± 2.9	22.0 ± 2.7	21.4 ± 2.7
Alcohol drinking *				
0	143,894 (67.6)	48,322 (82.6)	34,371 (66.2)	61,201 (59.6)
0–17	31,795 (14.9)	4916 (8.4)	10,040 (19.4)	16,839 (16.4)
18–35	19,054 (9.0)	2591 (4.4)	4346 (8.4)	12,117 (11.8)
>35	18,253 (8.5)	2676 (4.6)	3098 (6.0)	12,479 (12.2)
Consumption of fruits and vegetables				
No	133,093 (62.4)	35,190 (60.1)	30,055 (57.9)	67,848 (66.0)
Yes	80,128 (37.6)	23,393 (39.9)	21,836 (42.1)	34,899 (34.0)
Indoor air pollution				
No	35,605 (16.7)	11,523 (19.7)	9815 (18.9)	14,267 (13.9)
Yes	177,616 (83.3)	47,060 (80.3)	42,076 (81.1)	88,480 (86.1)
Region				
South	115,142 (54.1)	30,840 (52.6)	22,813 (44.0)	61,489 (60.0)
North	98,079 (45.9)	27,743 (47.4)	29,078 (56.0)	41,258 (40.0)
Urbanicity				
Urban	61,407 (28.9)	19,517 (33.3)	17,641 (34.0)	24,249 (23.6)
Rural	151,814 (71.1)	39,066 (66.7)	34,250 (66.0)	78,498 (76.4)

* 225 cases with missing value for alcohol drinking. BMI: body mass index.

**Table 2:** Hazard ratios (and 95% confidence interval, or CI) of mortality associated with 10-mmHg increase in baseline systolic blood pressure (SBP) and diastolic blood pressure (DBP) and with categories of blood pressure in baseline *. IHD: ischemic heart disease.

Cause of Death	No. of Death	Adjusted Model (1)	Adjusted Model (2)	Adjusted Model (3)
10-mmHg increase in baseline SBP and DBP				
SBP				
All-cause	52,795	1.05 (1.05–1.06)	1.07 (1.06–1.08)	1.07 (1.06–1.07)
Cardiovascular	18,833	1.15 (1.15–1.16)	1.16 (1.16–1.17)	1.16 (1.15–1.17)
IHD	3744	1.11 (1.09–1.13)	1.10 (1.08–1.12)	1.08 (1.06–1.10)
Stroke	11,288	1.19 (1.18–1.20)	1.20 (1.19–1.21)	1.20 (1.19–1.21)
DBP				
All-cause	52,795	1.10 (1.10–1.11)	1.12 (1.12–1.13)	1.12 (1.11–1.13)
Cardiovascular	18,833	1.27 (1.26–1.29)	1.28 (1.26–1.30)	1.27 (1.25–1.28)
IHD	3744	1.17 (1.14–1.20)	1.14 (1.11–1.18)	1.11 (1.08–1.15)
Stroke	11,288	1.35 (1.33–1.37)	1.36 (1.34–1.38)	1.35 (1.32–1.37)
categories of blood pressure in baseline ^#^				
All-cause	52,795			
Prehypertension	26,533	1.09 (1.07–1.11)	1.10 (1.07–1.13)	1.08 (1.05–1.11)
Hypertension	10,809	1.30 (1.26–1.33)	1.38 (1.34–1.43)	1.35 (1.31–1.40)
Cardiovascular	18,833			
Prehypertension	9261	1.39 (1.34–1.45)	1.38 (1.32–1.45)	1.31 (1.25–1.38)
Hypertension	4942	2.15 (2.05–2.25)	2.21 (2.09–2.33)	2.07 (1.95–2.18)
IHD	3744			
Prehypertension	1933	1.33 (1.22–1.45)	1.26 (1.14–1.40)	1.14 (1.03–1.27)
Hypertension	906	1.79 (1.62–1.99)	1.68 (1.48–1.89)	1.47 (1.30–1.66)
Stroke	11,288			
Prehypertension	5435	1.56 (1.48–1.65)	1.56 (1.46–1.66)	1.46 (1.37–1.55)
Hypertension	3161	2.70 (2.54–2.87)	2.76 (2.57–2.97)	2.51 (2.33–2.70)

* Model 1: adjusted for age; Model 2: model 1 + individual-level covariates; Model 3: model 2 + area-level covariates (urban/rural, region); ^#^ normal blood pressure as the reference in categories of blood pressure in baseline.

**Table 3:** Hazard ratios (and 95% CI) of mortality according to pack years, smoking status, smoking years, and packs per day *. HR: heart rate, CVD: cardiovascular disease.

	No. of Death	HR	No. of Death	HR	No. of Death	HR	*p* for Trend
	**0**		**pack years 0.1–19**		**pack years ≥ 20**		
All-cause	12,295	1	9097	1.15 (1.12–1.18)	27,418	1.21 (1.18–1.24)	<0.0001
CVD	4586	1	3324	1.14 (1.09–1.20)	9370	1.14 (1.10–1.19)	<0.0001
IHD	963	1	720	1.17 (1.06–1.30)	1825	1.22 (1.13–1.33)	<0.0001
Stroke	2661	1	2015	1.15 (1.08–1.22)	5540	1.14 (1.08–1.19)	<0.0001
	**Never**		**Former**		**Current**		
All-cause	13,059	1	4297	1.30 (1.25–1.34)	35,439	1.18 (1.15–1.20)	-
CVD	4905	1	1519	1.19 (1.12–1.26)	12,409	1.13 (1.10–1.17)	-
IHD	1005	1	376	1.35 (1.19–1.52)	2363	1.19 (1.10–1.29)	-
Stroke	2889	1	868	1.17 (1.08–1.26)	7531	1.13 (1.08–1.18)	-
	**0**		**years ≤ 20**		**years > 20**		
All-cause	12,470	1	2427	1.05 (1.00–1.10)	34,039	1.21 (1.18–1.23)	<0.0001
CVD	4653	1	823	1.10 (1.02–1.19)	11,860	1.15 (1.11–1.19)	<0.0001
IHD	986	1	196	1.13 (0.96–1.34)	2344	1.20 (1.11–1.30)	<0.0001
Stroke	2696	1	482	1.11 (1.00–1.23)	7071	1.15 (1.10–1.20)	<0.0001
	**0 < pack ≤ 1**		**1 < packs ≤ 2**		**packs > 2**		
All-cause	9389	1	17,618	1.05 (1.02–1.07)	9508	1.01 (0.98–1.04)	<0.0001
CVD	3494	1	6098	1.02 (0.98–1.06)	3102	0.96 (0.92–1.01)	<0.0001
IHD	765	1	1245	1.06 (0.97–1.16)	535	0.93 (0.83–1.05)	<0.0001
Stroke	2055	1	3632	1.03 (0.97–1.08)	1868	1.00 (0.93–1.06)	<0.0001

* All models was adjusted for age + individual-level covariates (marital status, educational level, smoke status, hypertension, alcohol drinking, units of alcohol per week, BMI (kg/m^2^), consumption of fresh fruit and vegetables and indoor air pollution) + area-level covariates (urbanity (urban vs. rural) and region (south vs. north)).

## Supplementary Materials

The following are available online at http://www.mdpi.com/1660-4601/15/5/1026/s1. Table S1: Joint effects of smoking and blood pressure level on the risk of all-cause mortality*; Table S2. Joint effects of smoking and blood pressure level on the risk of CVD mortality*. Table S3. Joint effects of smoking and blood pressure level on the risk of IHD mortality*. Table S4. Joint effects of smoking and blood pressure level on the risk of stroke mortality*.
