# Supplementary material for: Smoking, Blood Pressure, and Cardiovascular Disease Mortality in a Large Cohort of Chinese Men with 15 Years Follow-up
Source: Int J Environ Res Public Health. 2018 May 18;15(5):1026. doi: 10.3390/ijerph15051026 (PMC5982065; doi:10.3390/ijerph15051026)
Supplement: Supplementary file 1 [file ijerph-15-01026-s001.pdf]

International Journal of
Environmental Research and Public Health

Supplementary Materials

Smoking, Blood Pressure, and Cardiovascular Disease Mortality in a Large Cohort of Chinese Men with 15 Years Follow-up

Jibin Tan, Xiumin Zhang, Weihua Wang, Peng Yin, Xiaomin Guo and Maigeng Zhou

Table S1. Joint effects of smoking and blood pressure level on the risk of all-cause mortality*.

Pack years	normal	PH	HP
0	1.00	1.16(1.11-1.21)	1.43(1.35-1.51)
0.1-19	1.19(1.12-1.26)	1.30(1.24-1.37)	1.73(1.63-1.83)
≥20	1.28(1.23-1.35)	1.38(1.32-1.44)	1.69(1.61-1.77)

Table S2. Joint effects of smoking and blood pressure level on the risk of CVD mortality*.

Pack years	normal	PH	HP
0	1.00	1.33(1.22-1.44)	2.05(1.87-2.25)
0.1-19	1.10(0.98-1.22)	1.50(1.37-1.63)	2.55(2.31-2.81)
≥20	1.18(1.08-1.29)	1.52(1.40-1.64)	2.30(2.12-2.50)

Table S3. Joint effects of smoking and blood pressure level on the risk of IHD mortality*.

Pack years	normal	PH	HP
0	1.00	1.21(1.02-1.45)	1.49(1.22-1.82)
0.1-19	1.18(0.94-1.48)	1.35(1.12-1.62)	1.98(1.60-2.46)
≥20	1.30(1.08-1.56)	1.47(1.24-1.74)	1.78(1.48-2.14)

Table S4. Joint effects of smoking and blood pressure level on the risk of stroke mortality*.

Pack years	normal	PH	HP
0	1.00	1.41(1.26-1.58)	2.55(2.26-2.88)
0.1-19	1.09(0.94-1.26)	1.64(1.46-1.84)	3.07(2.69-3.49)
≥20	1.16(1.03-1.31)	1.65(1.49-1.84)	2.74(2.45-3.07)

*Hazard ratios against blood pressure levels (Normal = normal blood pressure, PH = prehypertension, HP = hypertension) and pack years of smoking (0, 0.1–19, ≥20) adjusted for age, educational level, marital status, indoor air pollution, consumption of fruit and vegetables, alcohol drinking, urbanicity, and region).

APPENDENCIES

Tables including HR (and CI) for all variables in a given model

Joint effects of smoking and blood pressure level

Table a1. Joint effects of smoking and blood pressure level on the risk of all-cause mortality*.

variable		Degree of Freedom	estimates	standard means	chisq	Sig.	HR	95% CI	
joint	1	1	0.17179	0.0291	34.8523	<.0001	1.187	1.122	1.257
joint	2	1	0.25052	0.02346	114.0087	<.0001	1.285	1.227	1.345
joint	3	1	0.14796	0.02337	40.0754	<.0001	1.159	1.108	1.214
joint	4	1	0.26349	0.02476	113.2903	<.0001	1.301	1.24	1.366
joint	5	1	0.32208	0.0217	220.2302	<.0001	1.38	1.323	1.44
joint	6	1	0.3567	0.02776	165.1383	<.0001	1.429	1.353	1.508
joint	7	1	0.54612	0.03079	314.6645	<.0001	1.727	1.625	1.834
joint	8	1	0.52368	0.02413	470.8854	<.0001	1.688	1.61	1.77
DRALC		1	−0.1016	0.01405	52.2597	<.0001	0.903	0.879	0.929
units		1	0.0025	0.000302	68.58	<.0001	1.003	1.002	1.003
BMI		1	−0.0241	0.00188	164.4302	<.0001	0.976	0.973	0.98
iap		1	0.4778	0.01908	627.3158	<.0001	1.613	1.553	1.674
edugroup		1	−0.20955	0.0134	244.4005	<.0001	0.811	0.79	0.833
MARRIED		1	−0.10876	0.01359	64.0631	<.0001	0.897	0.873	0.921
fvgroup		1	−0.1499	0.01143	172.1345	<.0001	0.861	0.842	0.88

test of null hypothesis: BETA=0			
test	chisq	df	Pr > chisq
likelihood ratio	2871.129	15	<.0001
score	2760.165	15	<.0001
Wald	2735.978	15	<.0001

Table a2. Joint effects of smoking and blood pressure level on the risk of CVD mortality*.

variable		DF	estimates	standard means	chisq	Sig.	HR	95% CI	
joint	1	1	0.09283	0.0551	2.8383	0.092	1.097	0.985	1.222
joint	2	1	0.16698	0.04404	14.3735	0.0001	1.182	1.084	1.288
joint	3	1	0.28386	0.04211	45.4438	<.0001	1.328	1.223	1.443
joint	4	1	0.40404	0.04422	83.5051	<.0001	1.498	1.374	1.633
joint	5	1	0.41643	0.0397	110.0063	<.0001	1.517	1.403	1.639
joint	6	1	0.71788	0.04645	238.9018	<.0001	2.05	1.872	2.245
joint	7	1	0.93428	0.04992	350.304	<.0001	2.545	2.308	2.807
joint	8	1	0.8334	0.0423	388.0931	<.0001	2.301	2.118	2.5
DRALC		1	−0.1169	0.02365	24.4375	<.0001	0.89	0.849	0.932
units		1	0.00353	0.000511	47.937	<.0001	1.004	1.003	1.005
BMI		1	0.00782	0.00304	6.6333	0.01	1.008	1.002	1.014
iap		1	0.55509	0.03271	288.031	<.0001	1.742	1.634	1.857
edugroup		1	−0.16446	0.02262	52.8427	<.0001	0.848	0.812	0.887
MARRIED		1	−0.12209	0.02226	30.0716	<.0001	0.885	0.847	0.925
fvgroup		1	−0.0929	0.01912	23.6029	<.0001	0.911	0.878	0.946

test of null hypothesis: BETA=0			
test	chisq	df	Pr > chisq
likelihood ratio	1679.388	15	<.0001
score	1708.052	15	<.0001
Wald	1671.714	15	<.0001

Table a3. Joint effects of smoking and blood pressure level on the risk of IHD mortality*.

variable		DF	estimates	standard means	chisq	Sig.	HR	95% CI	
joint	1	1	0.16357	0.11535	2.0109	0.1562	1.178	0.939	1.476
joint	2	1	0.26037	0.09468	7.5626	0.006	1.297	1.078	1.562
joint	3	1	0.19266	0.09022	4.5606	0.0327	1.212	1.016	1.447
joint	4	1	0.2995	0.0946	10.0226	0.0015	1.349	1.121	1.624
joint	5	1	0.38338	0.08554	20.0876	<.0001	1.467	1.241	1.735
joint	6	1	0.39791	0.102	15.2178	<.0001	1.489	1.219	1.818
joint	7	1	0.68475	0.10917	39.3393	<.0001	1.983	1.601	2.456
joint	8	1	0.57668	0.09329	38.2127	<.0001	1.78	1.483	2.137
DRALC		1	–0.17411	0.05423	10.3065	0.0013	0.84	0.755	0.934
units		1	0.00191	0.00131	2.1216	0.1452	1.002	0.999	1.004
BMI		1	0.01485	0.00649	5.2398	0.0221	1.015	1.002	1.028
iap		1	0.31792	0.06115	27.0299	<.0001	1.374	1.219	1.549
edugroup		1	0.03006	0.04623	0.4226	0.5156	1.031	0.941	1.128
MARRIED		1	–0.26991	0.04853	30.9368	<.0001	0.763	0.694	0.84
fvgroup		1	0.03273	0.04145	0.6233	0.4298	1.033	0.953	1.121

test of null hypothesis: BETA=0			
test	chisq	df	Pr > chisq
likelihood ratio	153.5132	15	<.0001
score	156.4693	15	<.0001
Wald	155.2092	15	<.0001

Table a4. Joint effects of smoking and blood pressure level on the risk of stroke mortality*.

variable		DF	estimates	standard means	chisq	Sig.	HR	95% CI	
joint	1	1	0.08596	0.07524	1.3051	0.2533	1.09	0.94	1.263
joint	2	1	0.14858	0.06086	5.9603	0.0146	1.16	1.03	1.307
joint	3	1	0.34673	0.05772	36.0854	<.0001	1.414	1.263	1.584
joint	4	1	0.49363	0.05986	68.0104	<.0001	1.638	1.457	1.842
joint	5	1	0.50333	0.05445	85.4606	<.0001	1.654	1.487	1.841
joint	6	1	0.93519	0.06202	227.4046	<.0001	2.548	2.256	2.877
joint	7	1	1.12019	0.06589	289.0214	<.0001	3.065	2.694	3.488
joint	8	1	1.00861	0.0573	309.8864	<.0001	2.742	2.451	3.068
DRALC		1	–0.09474	0.03039	9.7195	0.0018	0.91	0.857	0.965
units		1	0.00437	0.000644	45.9088	<.0001	1.004	1.003	1.006
BMI		1	0.01389	0.00393	12.462	0.0004	1.014	1.006	1.022
iap		1	0.66719	0.04513	218.528	<.0001	1.949	1.784	2.129
edugroup		1	–0.22624	0.02997	56.97	<.0001	0.798	0.752	0.846
MARRIED		1	–0.06275	0.02927	4.5954	0.0321	0.939	0.887	0.995
fvgroup		1	–0.14927	0.02518	35.1395	<.0001	0.861	0.82	0.905

test of null hypothesis: BETA=0			
test	chisq	df	Pr > chisq
likelihood ratio	1542.29	15	<.0001
score	1578.227	15	<.0001
Wald	1527.017	15	<.0001

*from Table a1 to Table a4, joint is the interaction between blood pressure and smoking status, joint 1–8 represent categories as follow in Table aa1. DRALC represents drinking alcohol regularly (0 = no (reference), 1 = yes), units represents the amount of drinking (drink, one drink is about 14g of pure

alcohol), BMI equals to weight (Kg)/height (m)2, iap represents indoor air pollution (0 = not exposed to indoor pollution (reference), 1 = exposed to indoor pollution), edugroup represents education level (0 = less than six years (reference), 1 = equal to or more than six years), married represents marital status (0 = not married (reference), 1 = married), fvgroup represents consumption of fruit and vegetables regularly (0 = no (reference), 1 = yes).

Table aa1. Categories of interaction between blood pressure and smoking status.

		Blood pressure		
		normal blood pressure	prehypertension	hypertension
Pack years	0	reference	Joint 3	Joint 6
	0.1–19	Joint1	Joint 4	Joint 7
	≥20	Joint 2	Joint 5	Joint 8

Pack years

Table b1. Pack years of smoking on the risk of all cause mortality#.

variable		DF	estimates	standard means	chisq	Sig.	HR	95% CI	
**pack years**	**1**	1	0.1405	0.01424	97.3866	<.0001	1.151	1.119	1.183
**pack years**	**2**	1	0.1897	0.01129	282.4784	<.0001	1.209	1.182	1.236
**hypertension1**		1	0.21795	0.00992	482.6476	<.0001	1.244	1.22	1.268
**DRALC**		1	−0.10129	0.01405	51.949	<.0001	0.904	0.879	0.929
**units**		1	0.00249	0.000302	67.8777	<.0001	1.002	1.002	1.003
**BMI**		1	−0.02355	0.00187	157.9332	<.0001	0.977	0.973	0.98
**iap**		1	0.48067	0.01908	634.9922	<.0001	1.617	1.558	1.679
**edugroup**		1	−0.20975	0.01341	244.7743	<.0001	0.811	0.79	0.832
**MARRIED**		1	−0.10834	0.01359	63.5683	<.0001	0.897	0.874	0.922
**fvgroup**		1	−0.14982	0.01143	171.906	<.0001	0.861	0.842	0.88

**test of null hypothesis: BETA=0**			
**test**	**chisq**	**df**	**Pr > chisq**
**likelihood ratio**	2829.231	10	<.0001
**score**	2712.749	10	<.0001
**Wald**	2692.885	10	<.0001

Table b2. Pack years of smoking on the risk of CVD #.

variable		DF	estimates	standard means	chisq	Sig.	HR	95% CI	
**pack years**	**1**	1	0.13477	0.02343	33.0809	<.0001	1.144	1.093	1.198
**pack years**	**2**	1	0.13398	0.01877	50.9305	<.0001	1.143	1.102	1.186
**hypertension1**		1	0.4686	0.01624	832.1268	<.0001	1.598	1.548	1.649
**DRALC**		1	−0.1176	0.02364	24.752	<.0001	0.889	0.849	0.931
**units**		1	0.00356	0.00051	48.8489	<.0001	1.004	1.003	1.005
**BMI**		1	0.00961	0.00303	10.0828	0.0015	1.01	1.004	1.016
**iap**		1	0.56076	0.03271	293.9562	<.0001	1.752	1.643	1.868
**edugroup**		1	−0.16521	0.02263	53.2801	<.0001	0.848	0.811	0.886
**MARRIED**		1	−0.1221	0.02226	30.0829	<.0001	0.885	0.847	0.925
**fvgroup**		1	−0.09336	0.01913	23.8203	<.0001	0.911	0.877	0.946

**test of null hypothesis: BETA=0**			
**test**	**chisq**	**df**	**Pr > chisq**
**likelihood ratio**	1552.076	10	<.0001
**score**	1547.211	10	<.0001
**Wald**	1525.77	10	<.0001

Table b3. Pack years of smoking on the risk of IHD #.

variable		DF	estimates	standard means	chisq	Sig.	HR	95% CI	
**pack years**	**1**	1	0.15967	0.0508	9.8801	0.0017	1.173	1.062	1.296
**pack years**	**2**	1	0.20115	0.04162	23.3588	<.0001	1.223	1.127	1.327
**hypertension1**		1	0.25275	0.03632	48.4393	<.0001	1.288	1.199	1.383
**DRALC**		1	−0.17455	0.05422	10.3643	0.0013	0.84	0.755	0.934
**units**		1	0.00192	0.00131	2.1615	0.1415	1.002	0.999	1.004
**BMI**		1	0.01566	0.00647	5.8525	0.0156	1.016	1.003	1.029
**iap**		1	0.32104	0.06115	27.5603	<.0001	1.379	1.223	1.554
**edugroup**		1	0.02808	0.04624	0.3689	0.5436	1.028	0.939	1.126
**MARRIED**		1	−0.26996	0.04852	30.9534	<.0001	0.763	0.694	0.84
**fvgroup**		1	0.03329	0.04146	0.6446	0.422	1.034	0.953	1.121

**test of null hypothesis: BETA=0**			
**test**	**chisq**	**df**	**Pr > chisq**
**likelihood ratio**	145.298	10	<.0001
**score**	146.9133	10	<.0001
**Wald**	146.3002	10	<.0001

Table b4. Pack years of smoking on the risk of Stroke #.

variable		DF	estimates	standard means	chisq	Sig.	HR	95% CI	
**pack years**	**1**	1	0.13782	0.03033	20.6479	<.0001	1.148	1.082	1.218
**pack years**	**2**	1	0.1292	0.02454	27.722	<.0001	1.138	1.084	1.194
**hypertension1**		1	0.58096	0.02108	759.8875	<.0001	1.788	1.715	1.863
**DRALC**		1	−0.09636	0.03038	10.0633	0.0015	0.908	0.856	0.964
**units**		1	0.00443	0.000644	47.2566	<.0001	1.004	1.003	1.006
**BMI**		1	0.01645	0.00392	17.5759	<.0001	1.017	1.009	1.024
**iap**		1	0.67419	0.04513	223.187	<.0001	1.962	1.796	2.144
**edugroup**		1	−0.22685	0.02999	57.2273	<.0001	0.797	0.752	0.845
**MARRIED**		1	−0.06302	0.02927	4.6362	0.0313	0.939	0.887	0.994
**fvgroup**		1	−0.15016	0.02519	35.5235	<.0001	0.861	0.819	0.904

**test of null hypothesis: BETA=0**			
**test**	**chisq**	**df**	**Pr > chisq**
**likelihood ratio**	1383.749	10	<.0001
**score**	1383.016	10	<.0001
**Wald**	1353.922	10	<.0001

#from Table b1 to Table b4, pack years 1 = 0.1–19 pack years, pack years 2 = equal to or more than 20 pack years, hypertension1 = (0 = normotensive, 1 = hypertension), DRALC represents drinking alcohol regularly (0 = no (reference), 1 = yes), units represents the amount of drinking (drink, one drink is about 14g of pure alcohol), BMI equals to weight (Kg)/height (m)2, iap represents indoor air pollution (0 = not exposed to indoor pollution (reference), 1 = exposed to indoor pollution), edugroup represents education level (0 = less than six years (reference), 1 = equal to or more than six years), married represents marital status (0 = not married (reference), 1 = married), fvgroup represents consumption of fruit and vegetables regularly (0 = no (reference), 1 = yes).

10mmHg increase in baseline SBP and DBP

Table c1. 10mmHg increase in baseline SBP on the risk of all cause mortality.

variable	DF	estimates	standard means	chisq	Sig.	HR	95% CI

**sbp**	1	0.06627	0.00255	674.9832	<.0001	1.069	1.063	1.074
**smk_status**	1	−0.09745	0.01735	31.5363	<.0001	0.907	0.877	0.939
**packyear**	1	0.000182	0.000198	0.8479	0.3571	1	1	1.001
**DRALC**	1	−0.1035	0.01475	49.2331	<.0001	0.902	0.876	0.928
**units**	1	0.00263	0.000309	72.6921	<.0001	1.003	1.002	1.003
**BMI**	1	−0.03125	0.00214	212.3536	<.0001	0.969	0.965	0.973
**iap**	1	0.51811	0.02238	536.0381	<.0001	1.679	1.607	1.754
**edugroup**	1	−0.21661	0.01527	201.1181	<.0001	0.805	0.781	0.83
**MARRIED**	1	−0.10509	0.016	43.1386	<.0001	0.9	0.872	0.929
**fvgroup**	1	−0.14386	0.01283	125.6359	<.0001	0.866	0.844	0.888

Table c2. 10mmHg increase in baseline SBP on the risk of CVD.

variable	DF	estimates	standard means	chisq	Sig.	HR	95% CI	
**sbp**	1	0.14821	0.00395	1405.643	<.0001	1.16	1.151	1.169
**smk_status**	1	−0.0434	0.02938	2.1818	0.1397	0.958	0.904	1.014
**packyear**	1	−0.00013	0.000337	0.1434	0.7049	1	0.999	1.001
**DRALC**	1	−0.12428	0.02479	25.1285	<.0001	0.883	0.841	0.927
**units**	1	0.00387	0.000514	56.6805	<.0001	1.004	1.003	1.005
**BMI**	1	0.00098	0.00349	0.0789	0.7788	1.001	0.994	1.008
**iap**	1	0.57647	0.03883	220.3788	<.0001	1.78	1.649	1.92
**edugroup**	1	−0.17674	0.02593	46.4546	<.0001	0.838	0.796	0.882
**MARRIED**	1	−0.11085	0.02624	17.8491	<.0001	0.895	0.85	0.942
**fvgroup**	1	−0.10215	0.02169	22.1807	<.0001	0.903	0.865	0.942

Table c3. 10mmHg increase in baseline SBP on the risk of IHD.

variable	DF	estimates	standard means	chisq	Sig.	HR	95% CI	
**sbp**	1	0.07964	0.00961	68.6395	<.0001	1.083	1.063	1.103
**smk_status**	1	−0.09669	0.06136	2.483	0.1151	0.908	0.805	1.024
**packyear**	1	8.93E-05	0.000784	0.013	0.9093	1	0.999	1.002
**DRALC**	1	−0.19643	0.0571	11.8357	0.0006	0.822	0.735	0.919
**units**	1	0.00366	0.00128	8.1269	0.0044	1.004	1.001	1.006
**BMI**	1	0.00769	0.00761	1.0213	0.3122	1.008	0.993	1.023
**iap**	1	0.34863	0.07266	23.0239	<.0001	1.417	1.229	1.634
**edugroup**	1	0.02927	0.05339	0.3005	0.5836	1.03	0.927	1.143
**MARRIED**	1	−0.22092	0.05828	14.3689	0.0002	0.802	0.715	0.899
**fvgroup**	1	0.04352	0.04729	0.8471	0.3574	1.044	0.952	1.146

Table c4. 10mmHg increase in baseline SBP on the risk of stroke.

variable	DF	estimates	standard means	chisq	Sig.	HR	95% CI	
**sbp**	1	0.179	0.00494	1314.474	<.0001	1.196	1.185	1.208
**smk_status**	1	−0.03163	0.03864	0.67	0.4131	0.969	0.898	1.045
**packyear**	1	0.000147	0.000436	0.1128	0.737	1	0.999	1.001
**DRALC**	1	−0.10151	0.03165	10.2895	0.0013	0.903	0.849	0.961
**units**	1	0.00447	0.000642	48.5895	<.0001	1.004	1.003	1.006
**BMI**	1	0.004	0.00449	0.7918	0.3735	1.004	0.995	1.013
**iap**	1	0.69012	0.05359	165.8388	<.0001	1.994	1.795	2.215
**edugroup**	1	−0.25556	0.03421	55.7927	<.0001	0.774	0.724	0.828
**MARRIED**	1	−0.04426	0.03416	1.6779	0.1952	0.957	0.895	1.023
**fvgroup**	1	−0.19578	0.02856	46.9955	<.0001	0.822	0.777	0.87

Table c5. 10mmHg increase in baseline DBP on the risk of all cause mortality.

variable	DF	estimates	standard means	chisq	Sig.	HR	95% CI	
**dbp**	1	0.11449	0.00423	733.1447	<.0001	1.121	1.112	1.131
**smk_status**	1	−0.10174	0.01735	34.3752	<.0001	0.903	0.873	0.935
**packyear**	1	0.000256	0.000198	1.6771	0.1953	1	1	1.001
**DRALC**	1	−0.10613	0.01475	51.747	<.0001	0.899	0.874	0.926
**units**	1	0.00266	0.000309	74.0611	<.0001	1.003	1.002	1.003
**BMI**	1	−0.03144	0.00214	215.0235	<.0001	0.969	0.965	0.973
**iap**	1	0.52118	0.02239	541.7376	<.0001	1.684	1.612	1.76
**edugroup**	1	−0.21374	0.01528	195.6373	<.0001	0.808	0.784	0.832
**MARRIED**	1	−0.10264	0.016	41.1375	<.0001	0.902	0.875	0.931
**fvgroup**	1	−0.13722	0.01284	114.1994	<.0001	0.872	0.85	0.894

Table c6. 10mmHg increase in baseline DBP on the risk of CVD.

variable	DF	estimates	standard means	chisq	Sig.	HR	95% CI	
**dbp**	1	0.2363	0.00683	1197.366	<.0001	1.267	1.25	1.284
**smk_status**	1	−0.05435	0.02938	3.4225	0.0643	0.947	0.894	1.003
**packyear**	1	0.000045	0.000335	0.018	0.8934	1	0.999	1.001
**DRALC**	1	−0.12977	0.02479	27.4009	<.0001	0.878	0.837	0.922
**units**	1	0.004	0.000514	60.6706	<.0001	1.004	1.003	1.005
**BMI**	1	0.00254	0.00349	0.5295	0.4668	1.003	0.996	1.009
**iap**	1	0.58826	0.03888	228.8945	<.0001	1.801	1.669	1.943
**edugroup**	1	−0.16746	0.02596	41.5971	<.0001	0.846	0.804	0.89
**MARRIED**	1	−0.11234	0.02623	18.3463	<.0001	0.894	0.849	0.941
**fvgroup**	1	−0.08973	0.02168	17.1232	<.0001	0.914	0.876	0.954

Table c7. 10mmHg increase in baseline DBP on the risk of IHD.

variable	DF	estimates	standard means	chisq	Sig.	HR	95% CI	
**dbp**	1	0.10517	0.01614	42.4811	<.0001	1.111	1.076	1.147
**smk_status**	1	−0.10252	0.06134	2.7937	0.0946	0.903	0.8	1.018
**packyear**	1	0.00019	0.000783	0.059	0.8082	1	0.999	1.002
**DRALC**	1	−0.20021	0.05708	12.3033	0.0005	0.819	0.732	0.915
**units**	1	0.0038	0.00128	8.8116	0.003	1.004	1.001	1.006
**BMI**	1	0.01015	0.0076	1.7817	0.1819	1.01	0.995	1.025
**iap**	1	0.35786	0.07269	24.2401	<.0001	1.43	1.24	1.649
**edugroup**	1	0.03329	0.05343	0.3883	0.5332	1.034	0.931	1.148
**MARRIED**	1	−0.22424	0.05826	14.8152	0.0001	0.799	0.713	0.896
**fvgroup**	1	0.04983	0.04727	1.1115	0.2918	1.051	0.958	1.153

Table c8. 10mmHg increase in baseline DBP on the risk of stroke.

variable	DF	estimates	standard means	chisq	Sig.	HR	95% CI	
**dbp**	1	0.29652	0.0086	1188.616	<.0001	1.345	1.323	1.368
**smk_status**	1	−0.04367	0.03864	1.2773	0.2584	0.957	0.887	1.033
**packyear**	1	0.000328	0.000433	0.5724	0.4493	1	0.999	1.001
**DRALC**	1	−0.10641	0.03164	11.3095	0.0008	0.899	0.845	0.957
**units**	1	0.00454	0.000643	49.9913	<.0001	1.005	1.003	1.006
**BMI**	1	0.00526	0.00449	1.3692	0.242	1.005	0.996	1.014
**iap**	1	0.70419	0.05365	172.2549	<.0001	2.022	1.82	2.246
**edugroup**	1	−0.24389	0.03427	50.6638	<.0001	0.784	0.733	0.838
**MARRIED**	1	−0.04709	0.03415	1.902	0.1679	0.954	0.892	1.02
**fvgroup**	1	−0.1826	0.02855	40.9095	<.0001	0.833	0.788	0.881

#from Table c1 to Table c8, pack years 1 = 0.1–19 pack years, pack years 2 = equal to or more than 20 pack years, smk_status = (0 = never smoked, 1 = formerly smoked, 2 = currently smoke), DRALC represents drinking alcohol regularly (0 = no (reference), 1 = yes), units represents the amount of drinking (drink, one drink is about 14g of pure alcohol), BMI equals to weight (Kg)/height (m)2, iap represents indoor air pollution (0 = not exposed to indoor pollution (reference), 1 = exposed to indoor pollution), edugroup represents education level (0 = less than six years (reference), 1 = equal to or more than six years), married represents marital status (0 = not married (reference), 1 = married), fvgroup represents consumption of fruit and vegetables regularly (0 = no (reference), 1 = yes).
